# Micro and Nano Smart Composite Films Based on Copper-Iodine Coordination Polymer as Thermochromic Biocompatible Sensors

**DOI:** 10.3390/polym11061047

**Published:** 2019-06-15

**Authors:** Javier Conesa-Egea, Alberto Moreno-Vázquez, Vanesa Fernández-Moreira, Yolanda Ballesteros, Milagros Castellanos, Félix Zamora, Pilar Amo-Ochoa

**Affiliations:** 1 Departamento de Química Inorgánica, Universidad Autónoma de Madrid, 28049 Madrid, Spain; javier.conesa@estudiante.uam.es (J.C.-E.); alberto.morenov@estudiante.uam.es (A.M.-V.); felix.zamora@uam.es (F.Z.); 2 Condensed Matter Physics Center (IFIMAC), Universidad Autónoma de Madrid, 28049 Madrid, Spain; 3 Departamento de Química Inorgánica, Instituto de Síntesis Química y Catálisis Homogénea (ISQCH), CSIC-Universidad de Zaragoza, 50009 Zaragoza, Spain; vanefm78@gmail.com; 4 Instituto de Investigación Tecnológica, Departamento de Ingeniería Mecánica, Universidad Pontificia Comillas, 28015 Madrid, Spain; yballesteros@icai.comillas.edu; 5 Instituto Madrileño de Estudios Avanzados en Nanociencia (IMDEA Nanociencia), Centro Nacional de Biotecnología (CNB-CSIC) Associated Unit, 28049 Madrid, Spain; milagros.castellanos@imdea.org; 6 Institute for Advanced Research in Chemical Sciences (IAdChem), Universidad Autónoma de Madrid, 28049 Madrid, Spain

**Keywords:** coordination polymers, smart materials, functional composites, flexible composites, stimuli-responsive materials

## Abstract

Herein is presented the preparation and characterization of a composite material obtained by the combination of nanosheets of a coordination polymer (CP) based on the copper(I)-I double chain with response to temperature and pressure with polylactic acid (PLA) as biodegradable organic matrix. The new films of composite materials are generated using a simple and low-cost method and can be created with long lateral dimensions and thicknesses ranging from a few microns to a few nanometers. Studies show that the new material maintains the optical response versus the temperature, while the elasticity and flexibility of the PLA totally quenches the response to pressure previously observed for the CP. This new material can act as a reversible sensor at low temperatures, thanks to the flexibility of the copper(I)-iodine chain that conforms the CP. The addition of CP to the PLA matrix reduces the elastic modulus and ultimate elongation of the organic matrix, although it does not reduce its tensile strength.

## 1. Introduction

Coordination polymers (CPs) are a family of compounds formed by combination of two or more building blocks that bring together metal entities with organic and/or inorganic molecules; these building blocks make use of coordination bonds to form the CP’s main network. They can be designed following simple principles of modular chemistry to form structures with different dimensionality (1D, 2D, or 3D) and a wide variety of architectures; moreover, the selection of the building blocks can also modulate their physical properties. Indeed, among these properties, they can show emission, electrical conductivity, magnetism, and/or porosity [1,2,3,4,5] or a combination of these properties to originate multifunctional materials. Therefore, they are candidates for different technological applications in fields [6] such as sensors of pollutants such as heavy metals or toxic gases in water, gases separation and entrapment [7], or catalysts [8].

**Figure 1:**
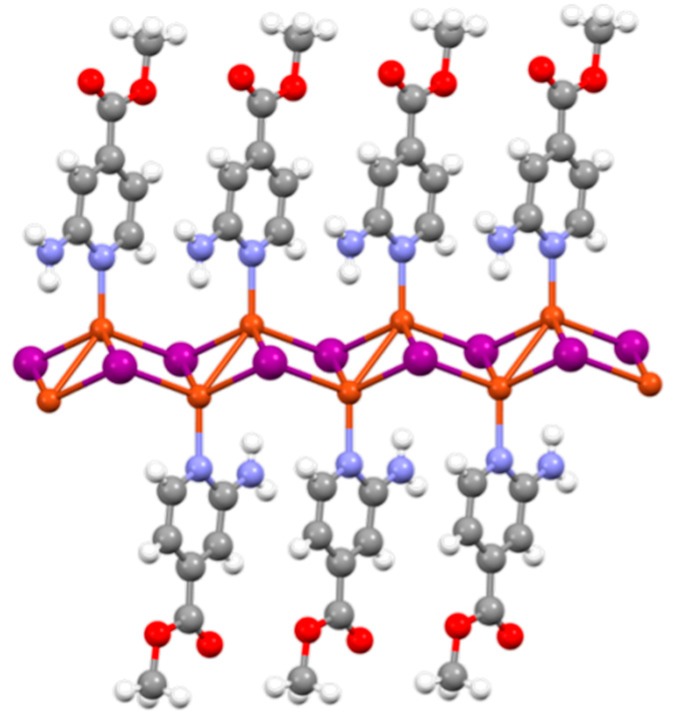
Example of a one-dimensional coordination polymer based on a copper(I)-iodide double chain. The represented coordination polymer (CP) is **[CuI(NH_2_-MeIN)_2_]_n_** (NH_2_-MeIN = methyl 2-aminoisonicotinate) [22]. Grey: C; white: H; blue: N; red: O; orange: Cu; purple: I.

Currently, their structural, chemical, and physical properties have attracted attention towards these materials, so the number of coordination polymers published to date is huge. Within CPs, there is a subfamily formed by the combination of CuI and N-donor organic ligands, i.e., pyridine, pyrimidine, or pyrazine derivatives, with functional groups capable of recognizing several molecules. They can form one- or two-dimensional structures with a common structural motif present in their skeleton—the double copper(I)-I chain [9,10]. They show interesting electrical and optical properties [11,12,13,14,15], but what is even more important is that these double copper(I)-I chains are extremely sensitive to external chemical or/and physical stimuli such as the presence of gases [16], temperature [17], or pressure [18,19]. These stimuli can cause slight structural changes along the double copper(I)-I chains which provoke significant changes in their physical properties, e.g., luminescence and/or conductivity [17,19]. In addition, these changes are reversible, which implies that these materials behave like small and flexible molecular springs [20] (Figure 1). In a few words, these CPs seem to be excellent candidates to produce new intelligent, stimuli-responsive materials. Other advantages of these copper(I)-Iodine double chain CPs are (1) easy synthesis, one-pot at room temperature; and (2) high insolubility in the reaction media which allows the direct production of nanostructures, e.g., nanofibers or nanosheets [21,22] using fast precipitation in poor solvents.

The fabrication of nanostructures of these CPs facilitates their integration within organic matrices to form new (multi)functional composites, or even stimuli-responsive composites, based on the hypothesis of the integration of the CP’s properties to the new composite.

The organic matrix must be selected considering the current need for plastic waste management, in which the partial or full replacement of bioresistant synthetic polymers by biodegradable and biocompatible polymers is highly recommended [23]. Another important advance in the manufacture of new composite materials is the ability to modulate their dimensions to create ultra-thin films. With this in mind, the fields of application of these new materials are extended, moving from material technologies to the fabrication of new nanodevices. Thus, the combination of nanoscale coordination polymers (NCPs) with interesting optical properties with suitable organic matrices to improve the mechanical properties in order to create films of composite materials and the ability to modulate their size using easy and low-cost methodologies are two important challenges that will allow a huge increase in their possible industrial applications.

In this work, we show an easy and simple preparation methodology of new smart composite thin films with interesting optical and mechanical properties, formed by the combination of nanosheets of the CP with formula **[Cu_2_I_2_(Apyz)]_n_** (Apyz = 2-aminopyrazine) with polylactic acid (PLA) as organic matrix, namely **[Cu_2_I_2_(Apyz)]_n_@PLA**. The new **[Cu_2_I_2_(Apyz)]_n_@PLA** composites have been obtained with different weight concentrations of **[Cu_2_I_2_(Apyz)]_n_** versus PLA: 1%, 4%, and 30% *w*/*w*.

The composites have been morphologically and structurally characterized using scanning electron microscopy (SEM) and atomic force microscopy (AFM), energy-dispersive X-ray spectroscopy (EDX), infrared spectroscopy (IR), and X-ray powder diffraction, confirming that the nanolayers of **[Cu_2_I_2_(Apyz)]_n_** are homogeneously distributed in the thin-film composite, with centimeter dimensions and controllable thicknesses from microns to nanometers. We have also checked the thermal decomposition of the films, this one being very similar to that of their independent components, and their mechanical and thermoluminescent properties. Remarkably, the composite films show a yellow emission which changes with temperature.

## 2. Materials and Methods

All reagents and solvents purchased were used without further purification. **[Cu_2_I_2_(Apyz)]_n_** nanosheets were prepared as previously reported [19]. PLA ([CH(CH_3_)COO]_n_) with natural biopolymer quality (3 mm nominal granule diameter) was purchased from Goodfellow via Sigma-Aldrich (Coraopolis, PA, USA).

IR spectra were recorded with a PerkinElmer 100 spectrophotometer (PerkinElmer Spain, Tres Cantos (Madrid), Spain) using a universal ATR sampling accessory from 4000 to 650 cm^−1^. Powder X-ray diffraction data was collected using a PANalytical X’Pert PRO diffractometer (Malvern PANalaytical Spain, Madrid, Spain) with θ/2θ primary monochromator and X’Celerator fast detector. The samples have been analyzed with scanning θ/2θ. Thermogravimetric analyses (TGA) were carried out on a TA Instruments Q500 thermobalance (New Castle, DE, USA) oven with a Pt sample holder and N_2_ as purge gas, at a flow rate of 90 mL/min; the samples were heated from 25 to 1000 °C at a rate of 10 °C/min.

The transparency of the **[Cu_2_I_2_(Apyz)]_n_@PLA** thin films was measured by UV–visible spectroscopy using an Agilent 8452 diode array spectrophotometer (Santa Clara, CA, USA) over the solid samples. The spectra were collected in the wavelength range between 190 and 1100 nm.

Scanning Electron Microscopy (SEM) images were taken in a Philips XL 30 S-FEG electron microscope (Amsterdam, The Netherlands), applying an electron beam of 300 μA intensity and 10.0 kV potential, at a pressure of 10^−7^ Pa. To obtain reproducible results, very flat substrates were used with precisely functionalized samples. Doped SiO_2_ surfaces were sonicated in ultrasound bath (Elma [Singen, Germany], 37 kHz, 380 W) for 15 min in acetone and 15 min in 2-propanol, and then dried under an Argon flow. After sample preparation, the surfaces were metallized with a 10 nm thick Cr layer, at a pressure of 10^−3^ Pa. SEM-EDX and EDX spectra were taken in a Hitachi S-3000N microscope (Chiyoda (Tokyo), Japan) with an ESED detector coupled to an INCAx-sight EDX analyzer (Oxford Instruments, Abingdon (Oxfordshire), United Kingdom). For this technique, samples were metallized with a 15 nm thick Au layer, at a pressure of 10^−3^ Pa. 

Atomic Force Microscopy (AFM) images were registered in a Nanotec Electronica microscope (Tres Cantos (Madrid), Spain), at room temperature and in an open atmosphere, using Olympus cantilevers (Madrid, Spain) with a constant nominal force of 0.75 N/m. Images were processed by the use of the WSxM program (Nanotec Electronica, Tres Cantos (Madrid), Spain) [24,25].

The mechanical properties of the samples of **[Cu_2_I_2_(Apyz)]_n_@PLA** films with **[Cu_2_I_2_(Apyz)]_n_** weight concentrations of 1%, 4%, and 30% were performed using the universal testing machine IBTH 500 (SAE Ibertest^®^, Daganzo de Arriba (Madrid), Spain) equipped with a 500 N load cell. Specimens for tensile testing were prepared with films of 75 mm long and 25 mm wide, within a thickness range between 25 and 140 µm. In order to determine the thickness of the films an Olympus GX51 microscope (Madrid, Spain) with LCmicro for Windows was used, doing 4 measurements at the edge of each of the samples (Figure S9). Additionally, a QuaNix 7500 coating thickness measurer (Masking Máscaras y Sistemas de Protección SL, Sant Fruitós de Bages (Barcelona), Spain) based on Eddy current was employed to check that the thickness in the edge and in the middle of the samples was the same.

A double-sided adhesive tape (Tesa^®^, Hamburg, Germany) was adhered to the ends of all the samples in order to prevent the clamping jaws from causing damage in the samples during the test (Figure S8). The initial separation between tapes, as well as the initial separation between clamping jaws, was 30mm. This length will be the reference length to calculate the final elongation (%). Three different measurements were carried out for each composite sample. 

The stress-strain curves for each specimen was obtained at a constant crosshead rate of 5mm·min^−1^ and the Tensile Strength (TS), Young Modulus (E), total elongation and plastic elongation of the samples were calculated. The values shown in Table S2 and Figures S9–11 correspond to an average of three measurements for each sample and the bars indicate the standard deviation. Statistical analysis was performed using IBM SPSS Statistic 24 for Windows (SPSS Inc. Chicago, IL, USA) using repeated measures ANOVA and Scheffe post hoc analysis. A *p*-value of less than 0.05 was indicative of statistically significant differences.

The rugosimeter device is a CARL ZEISS rugosimeter (Oberkochen, Germany) model SURFCOM 1500SD2.

### 2.1. Cell Culture and Viability Studies

MCF7 (ATCC^®^ HTB-22™) and MDA-MB-231 (ATCC^®^ HTB-26™) cells were cultured in DMEM High glucose medium supplemented with 10% FBS, 1% streptomycin-penicillin and 1% l-glutamine (all were purchased from VWR, Radnor, PA, USA) at 37 °C in a Binder CB210 incubator (Tuttlingen, Germany, 5% CO_2_). All the procedures were performed inside a laminar flow hood Telstar CV-30/70 (Terrassa (Barcelona), Spain). Cells were grown in 24-well plates (40,000 cells/well) 24 h prior treatment with the **[Cu_2_I_2_(Apyz)]_n_@PLA-4%** (2, 10, and 20 mg respectively) or independently with **[Cu_2_I_2_(Apyz)]_n_** (800, 400, and 80 µg respectively) or PLA (40 µg). In the case of the **[Cu_2_I_2_(Apyz)]_n_@PLA-4%** the films were cut if necessary, to fit the well dimensions. **[Cu_2_I_2_(Apyz)]_n_** was resuspended in 0.4 ml of medium per well and PLA was added directly to the culture medium.

### 2.2. AlamarBlue Viability Assay

A stock solution of resazurin sodium salt (Sigma-Aldrich, St. Louis, MO, USA) (1 mg/mL) in PBS was diluted 1% (*v*/*v*) in complete DMEM medium and added to the cells. After 3 h in the incubator (37 °C), the fluorescence of 100 µl of supernatant was measured at 25 °C in a plate reader Synergy H4 Hybrid reader (BioTEK, Winooski, VT, USA), λex = 550 nm, λem = 590 nm. The intensity data was processed using the following Equation:

% Cell viability = [(Sample data−Negative c.)÷(Positive c. −Negative c.)] ×100

The positive control corresponds to untreated cells. A resazurin solution without cells was used as negative control. Statistical significance of the data was analyzed using the One-way ANOVA Tukey test implemented in R software.

### 2.3. Synthesis of **[Cu_2_I_2_(Apyz)]_n_@PLA** Films

The films were prepared by drop-casting method. PLA was doped with **[Cu_2_I_2_(Apyz)]_n_** in 1, 4, and 30 wt %. A 4.0 wt % solution of PLA in CHCl_3_ and the solid **[Cu_2_I_2_(Apyz)]_n_** were mixed in the different proportions and dispersed with sonication for 30 min, in an ultrasonic bath at 30 °C (37 kHz, 380 W). The resulting homogeneous suspensions (10 mL) were deposited on a square glass surface (10 cm × 10 cm). The composite materials were obtained after solvent evaporation from the previously prepared suspensions at 25 °C for 24 h.

### 2.4. Synthesis of **[Cu_2_I_2_(Apyz)]_n_@PLA** Ultra-thin Films

The films with nanometric thickness were prepared by spin-coating and dip-coating methods starting from a **[Cu_2_I_2_(Apyz)]_n_** and PLA suspension prepared the same way as outlined in the drop-casting deposition method. For spin-coating, 15 μL of the homogeneous suspension were deposited on a SiO_2_ spin-coater at 17,000 rpm for 30 sec. For dip-coating, a SiO_2_ surface was immersed in the suspension for 2 min and was raised at 10 mm/min. In both methods, the surfaces were dried with an Argon flow.

## 3. Results and Discussion

### 3.1. Synthesis and Characterization

**Figure 2:**
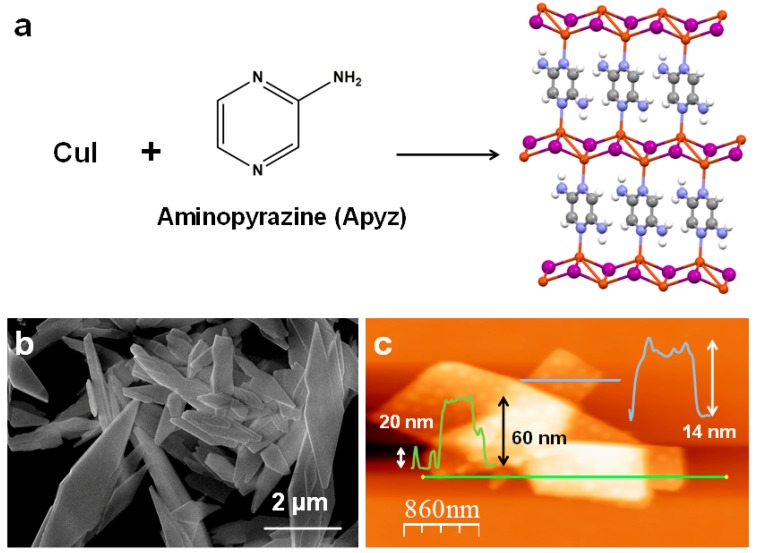
(**a**) Reaction that leads to the formation of **[Cu_2_I_2_(Apyz)I]_n_** and visualization of a 2D sheet of this CP (C: grey; H: white; N: blue; Cu: orange; I: purple). (**b**) FE-SEM image of **[Cu_2_I_2_(Apyz)]_n_** nanosheets. (**c**) AFM image of **[Cu_2_I_2_(Apyz)]_n_** nanosheets, with its height profiles across the lines.

The fabrication of composite films with nanometric thicknesses requires the incorporation of materials with nanometric dimensions. In this case, the two-dimensional coordination polymer **[Cu_2_I_2_(Apyz)]_n_** (Figure 2a) has been selected to prepare a smart composite material because of its optical properties i.e., reversible thermo- and mechanoluminescent response. **[Cu_2_I_2_(Apyz)]_n_** shows emission at 630 nm when recorded at 300 K that progressively blue-shifts as temperature drops, reaching 566 nm at 80 K with emission intensity about 30 times higher than that at 300 K. In addition, **[Cu_2_I_2_(Apyz)]_n_** presents a change of emission with the pressure. Thus, at low pressure (1 atm) it shows an emission band at 645 nm that experiences a red-shift to 690 nm when 5 GPa is applied with a gradual quenching of the intensity, causing the total vanishing of the emission when pressure reached about 8 GPa.

Interestingly, the adjustment of the synthetic conditions allows the preparation of nanosheets of **[Cu_2_I_2_(Apyz)]_n_** with nanometric thicknesses in a one-pot reaction (Figure 2b,c) [19].

**Figure 3:**
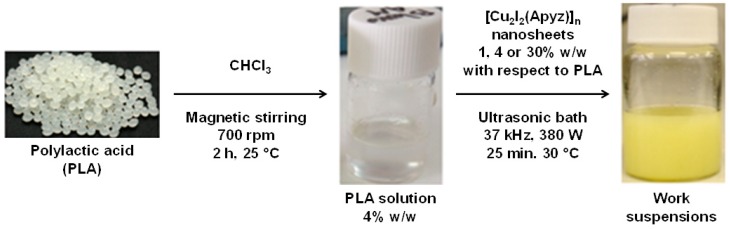
Scheme of the process that leads to the obtainment of homogeneous suspensions of **[Cu_2_I_2_(Apyz)]_n_@PLA**.

**Figure 4:**
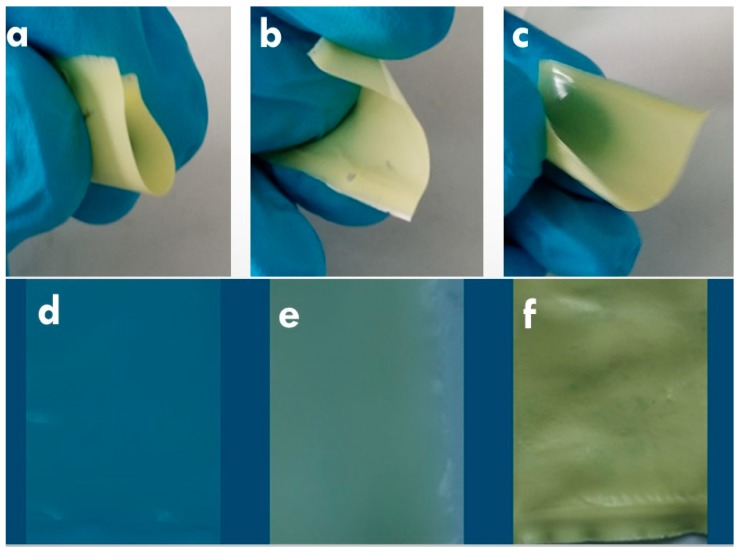
Flexibility of **[Cu_2_I_2_(Apyz)]_n_@PLA-30%** (**a**,**b**,**c**) and degree of visual transparency of the **[Cu_2_I_2_(Apyz)]_n_@PLA-1%** (**d**), **[Cu_2_I_2_(Apyz)]_n_@PLA-4%** (**e**) and **[Cu_2_I_2_(Apyz)]_n_@PLA-30%** (**f**).

The process to obtain the composite materials involves dissolving the PLA in chloroform, the addition of **[Cu_2_I_2_(Apyz)]_n_** (1, 4, and 30 wt %) and the sonication of the suspensions to form a homogeneous colloid (Figure 3) which upon slow solvent evaporation gives rise to the formation of flexible films of composite materials. The analytical, diffractometric, and spectroscopic analyses confirm the integration of **[Cu_2_I_2_(Apyz)]_n_** in the PLA matrix without alteration of its structure and composition (Figures S1 and S2). The obtained films are extremely flexible, and their transparency depends on the concentration of **[Cu_2_I_2_(Apyz)]_n_**: **[Cu_2_I_2_(Apyz)]_n_@PLA** films with 1% *w*/*w* of CP are translucent, films with 4% *w*/*w* of CP are almost opaque, and films with 30% *w*/*w* are fully opaque (Figure 4). A spectroscopic study carried out by UV–Visible spectroscopy confirms this finding, giving transparency values of 99% for PLA, 94% for **[Cu_2_I_2_(Apyz)]_n_@PLA** films with 1% *w*/*w*, 13% for films with 4% *w*/*w*, and 0% for films with 30% *w*/*w* (Figure S5).

The homogeneity of the **[Cu_2_I_2_(Apyz)]_n_** dispersed in the PLA matrix has been studied by FE-SEM (Figure S6) and SEM-EDX (Figure S7). High resolution SEM images give a first approximation of the high homogeneity of the **[Cu_2_I_2_(Apyz)]_n_@PLA** (**1%**, **4%**, and **30%**) films, showing surfaces with low roughness (*R*_a_ (mean arithmetic roughness) = 0.24, 0.23, and 0.20 µm and *R*_q_ (mean square roughness) = 0.31, 0.34, 0.29 µm, respectively) (Table S3), the roughness profile are also present in Figure S14.; moreover, the CP is well dispersed within the matrix, with no fibers poking out of it. In addition to this, the SEM-EDX data confirm both the homogeneity of the samples and the 1:1 proportion between copper and iodine in the composites.

### 3.2. Control of Composite Film Thickness

**Figure 5:**
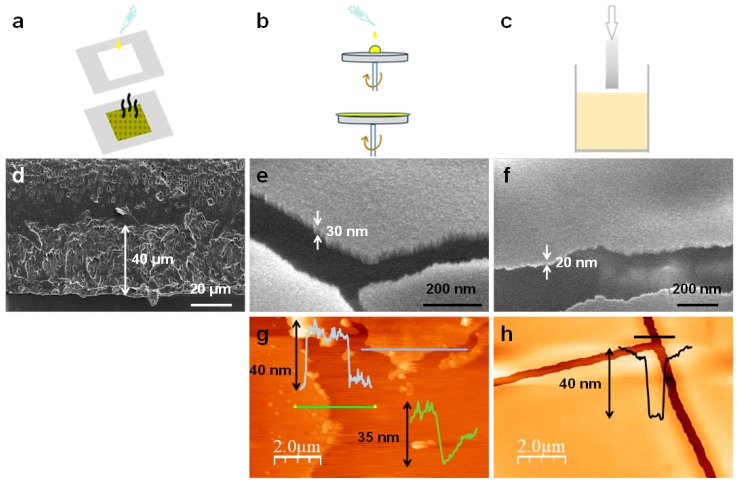
(**a**–**c**) Scheme of the deposition methods used to get **[Cu_2_I_2_(Apyz)]_n_@PLA** thin films: (**a**) Drop casting, (**b**) spin coating, (**c**) dip coating. (**d**) FE-SEM image of the **[Cu_2_I_2_(Apyz)]_n_@PLA** film with 30% *w*/*w* of **[Cu_2_I_2_(Apyz)]_n_** obtained using drop-casting as deposition method. (**e**,**g**) FE-SEM (**e**) and AFM (**g**) images of the **[Cu_2_I_2_(Apyz)]_n_@PLA** thin film with 30% *w*/*w* of **[Cu_2_I_2_(Apyz)]_n_** obtained using spin-coating as deposition method. (**f**,**h**) FE-SEM (**f**) and AFM (**h**) images of the **[Cu_2_I_2_(Apyz)]_n_@PLA** thin film with 30% *w*/*w* of **[Cu_2_I_2_(Apyz)]_n_** obtained using dip-coating as deposition method.

The thickness of film-composites can be controlled by changing the deposition method, from few microns for films prepared by drop-casting (Figure S6) to few tens of nanometers for thin films generated by spin-coating or dip-coating (Figure 5).

The thicknesses of the films have been studied by means of scanning electron microscopy (SEM) and atomic force microscopy (AFM) (Figure 5 and Figure S6). When the composite material is generated by drop-casting, the SEM images of the films supported on SiO_2_ show lateral thicknesses between 25 and 70 microns (Figure S6). However, the thicknesses of the films obtained by spin coating or deep coating observed by AFM show heights between 20 and 40 nanometers (Figure 5). This is in agreement with a previous study conducted on hybrid materials prepared from PVDF and the 1D CP **[CuI(NH_2_-MeIN)_2_]_n_** (Figure 1), namely, **[CuI(NH_2_-MeIN)_2_]_n_@PVDF** [22] where films prepared by a drop-casting method displayed thicknesses of around 40 microns and thin films generated by spin-coating or dip-coating approaches showed thicknesses between 25 and 60 nanometers.

The thicknesses of the films prepared via drop-casting appear in the ranges reported for similar hybrid materials, also known as mixed-matrix membranes (MMMs). Usually, the thicknesses reported for MMMs fall in the range between 35 to 100 microns [26,27]. On the other hand, the use of spin-coating has allowed the preparation of thin-films in which the thicknesses can be controlled with the centrifugation speed up to few nanometers. At smooth speeds, the resulting films can have thicknesses of 50 to 100 microns if the rotation speed is low [28,29], or in the rank between 300 and 470 nm at speeds up to 800 rpm [30]. However, at higher spinning rates (17,000 rpm) we have been able to obtain films with nanometric thicknesses (under 100 nm).

### 3.3. Tunable Luminescent Properties of **[Cu_2_I_2_(Apyz)]_n_@PLA** Films

One of the most relevant reasons to integrate CPs with organic matrices is to provide the new composites with the (multi)functionality of the selected CP. This includes the formation of composites with stimuli-responsive properties as those showed by the pristine CP. Therefore, once the new composites have been prepared as ultra-thin films, we have to evaluate their physical properties.

It is known that **[Cu_2_I_2_(Apyz)]_n_** is a thermochromic material showing a luminescence thermal response. Therefore, the thermoluminescence of the **[Cu_2_I_2_(Apyz)]_n_@PLA** composites has been studied and compared to that in the pristine CP.

**Figure 6:**
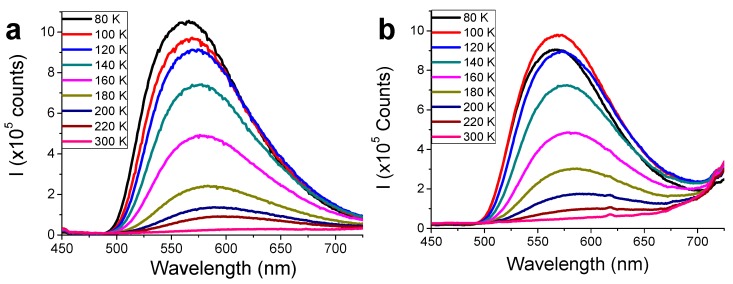
Thermal dependence of the luminescence spectra of **[Cu_2_I_2_(Apyz)]_n_** (**a**) and the **[Cu_2_I_2_(Apyz)]_n_@PLA** thin film with 30% *w*/*w* of **[Cu_2_I_2_(Apyz)]_n_** (**b**).

Figure 6 shows the luminescence spectra of **[Cu_2_I_2_(Apyz)]_n_@PLA** films (Figure 6b) and the pristine **[Cu_2_I_2_(Apyz)]_n_** (Figure 6a) with the temperature. We can observe that, while no significant changes in the emission bands are showed between the film composites and the pristine CP, the emission intensities of the **[Cu_2_I_2_(Apyz)]_n_@PLA** films decrease. As an example, **[Cu_2_I_2_(Apyz)]_n_@PLA** 30% *w*/*w* film and **[Cu_2_I_2_(Apyz)]_n_** show a very weak band emission centered at 630 nm at 300 K, which increases in intensity upon lowering the temperature (30 times higher from 300 to 100 K), and both materials show a progressive blue-shift. Since naked PLA does not show emission, the emission bands observed for the composites arise from metal-halide skeleton to ligand charge transfer triplet states (^3^(M+X)LCT) of the CP [19]. Therefore, these observations corroborate the potential use of the **[Cu_2_I_2_(Apyz)]_n_@PLA** thin-films as temperature sensors.

Additionally, since the emission of **[Cu_2_I_2_(Apyz)]_n_** is very sensitive to the pressure, we have studied the variations of the **[Cu_2_I_2_(Apyz)]_n_@PLA** thin-films’ emission with the pressure. It would be expected that **[Cu_2_I_2_(Apyz)]_n_@PLA** composite films displayed mechanical response as well as the thermal one. However, it has been found that the PLA matrix absorbs the effect of pressure. This has been observed in a naked-eye experiment where **[Cu_2_I_2_(Apyz)]_n_@PLA** films with the studied concentrations of **[Cu_2_I_2_(Apyz)]_n_** were compressed at pressures up to 6 GPa. Whereas the naked **[Cu_2_I_2_(Apyz)]_n_** would have lost its luminescence at 77 K, the hybrid materials kept their previous luminescent behavior. Although this fact rules out the possibility to use our composite as pressure sensors, they reveal the high resistance to impacts that these materials display.

### 3.4. Thermal Analysis

Neat PLA (Figure S3a) decomposes in a single step, taking place between 300 and 400 °C, (*T*_5%_ = 334 °C; *T*_max_ = 378 °C; see Table S1). On the other hand, the presence of **[Cu_2_I_2_(Apyz)]_n_** in the structure of the composite materials alters their way of decomposing (Figures S3b and S4, Table S1), the first step of the decomposition (at temperatures under 200 °C) occurs differently depending on the concentration of CP. In **[Cu_2_I_2_(Apyz)]_n_@PLA** films with 1% and 4% *w*/*w* of **[Cu_2_I_2_(Apyz)]_n_**, the ligand which is freed from the structure of the CP (0.20% and 0.80% of lost weight, respectively) drags part of the PLA with it; this has a remarkable effect on the on-set decomposition temperature (*T*_5%_), this one being 105 °C for **[Cu_2_I_2_(Apyz)]_n_@PLA 1%** and 118 °C for **[Cu_2_I_2_(Apyz)]_n_@PLA 4%**. On the other hand, in the film with 30% *w*/*w* (23% w.r.t. its total mass) only a 75% of the ligand is released under 200 °C; the rest of it disappears at about 300 °C; as a result, *T*_5%_ has a value of 245 °C for this material. Afterwards, all composite films decompose the same way: between 300 and 400 °C PLA degrades (this is why the temperatures at which the respective weight losses occur at maximum rate have close values). Finally, between 400 and 700 °C the CP finishes decomposing, delivering volatile Cu-I compounds [19]. The residual weight at the end of the degradation process is higher at higher initial CP concentrations.

### 3.5. Mechanical Stress Measurements of **[Cu_2_I_2_(Apyz)]_n_@PLA** Films

**Figure 7:**
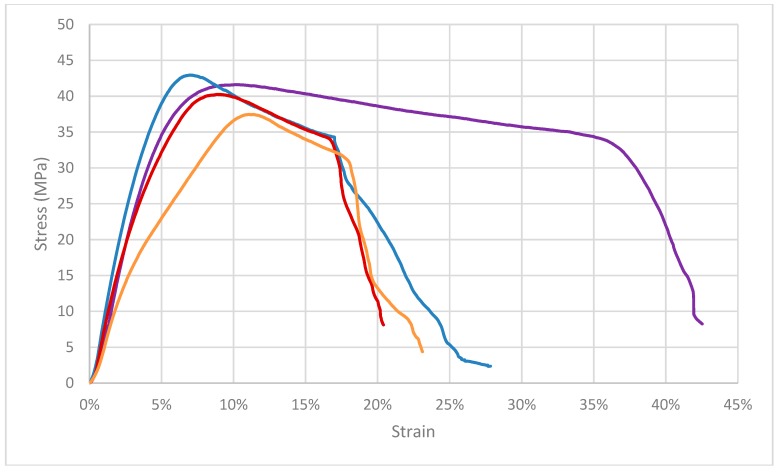
Stress-strain curves for pristine PLA (purple line) and **[Cu_2_I_2_(Apyz)]_n_@PLA** thin-films (blue line 1% *w*/*w*, red line 4% *w*/*w*, and orange line 30% *w*/*w*).

The mechanical behavior of pristine PLA and **[Cu_2_I_2_(Apyz)]_n_@PLA** thin-films are shown in Table S2 and Figure 7, Figure S10–12.

The addition of different amounts of **[Cu_2_I_2_(Apyz)]_n_** to the PLA matrix does not induce changes in the tensile strength of the films. Although there seems to be a slight increase in the ultimate tensile strength for the samples **[Cu_2_I_2_(Apyz)]_n_@PLA-1%** with respect to that observed for naked PLA (Figure S10), the statistical analysis indicates that there are no significant differences between them. A different behavior can be observed in the stiffness of the polymer (Figure S11), since the [**Cu_2_I_2_(Apyz)]_n_@PLA-1%** films showed an increase of 14% in the elastic modulus (E) when compared with PLA films (with a statistically significant difference, *p*-value = 0.017). The increase in the elastic modulus with this few amount of CP is indicative of good adhesion between polymer matrix and CP nanosheets. However, the addition of a high quantity of **[Cu_2_I_2_(Apyz)]_n_** (4% and 30%) gives rise to significant reductions in the Elastic Modulus of the films. The Young Modulus decreases a 16.6% for samples **[Cu_2_I_2_(Apyz)]_n_@PLA-4%** and a 27.3% for samples **[Cu_2_I_2_(Apyz)]_n_@PLA-30%** when compared with the samples **[Cu_2_I_2_(Apyz)]_n_@PLA-1%** films, being the differences of 4.9% and 17.0% when compared with the raw **PLA** (Figure S11). The decrease in the Young Modulus with the increase of the CP concentration should be related with the agglomeration of the CP and with a weak interfacial interaction between the CP and the organic matrix [23].

Moreover, Figure S12 shows that any amount of **[Cu_2_I_2_(Apyz)]_n_** in the composite films produces a decrease, close to 50%, in the total elongation of the film composites. During the tensile test the films undergo two different types of elongation—the elastic elongation which reverses when the applied load is released or when the film breaks, and the plastic or permanent deformation which remains when the material breaks. Figure S12 shows the total elongation, including plastic and elastic elongation. It is observed that increasing the content of **[Cu_2_I_2_(Apyz)]_n_** in the PLA composite from 1% *w*/*w* to 30% *w*/*w* produces no significant changes in the total elongation of the films. However, the samples exhibit a slight decrease in plastic deformation by increasing the amount of **[Cu_2_I_2_(Apyz)]_n_**, while the elastic elongation enhances due to its lower elastic modulus and, therefore, higher elasticity. Usually, MMMs based on porous or non-porous CP and a flexible organic matrix undergo a reduction in their ductility and ultimate tensile strength as the CP load increases [26,27]. Taking these facts into account, [**Cu_2_I_2_(Apyz)]_n_@PLA** composite films show a mechanical behavior very close to that of analogous MMMs.

### 3.6. Toxicity Tests of [Cu_2_I_2_(Apyz)]_n_@PLA 4% in MCF7 and MDA-MB-231 Tumoral Cells

In order to verify the degree of biocompatibility of the new composite material, toxicity tests of **[Cu_2_I_2_(Apyz)]_n_@PLA 4%** and **[Cu_2_I_2_(Apyz)]_n_** have been carried out on two types of adenocarcinoma cells, MCF7 (ATCC^®^ HTB-22 ™) and MDA-MB-231 (ATCC^®^ HTB-26 ™), comparing with the neat PLA.

For this purpose, cells of the two types have been incubated with the PLA, **[Cu_2_I_2_(Apyz)]_n_@PLA 4%** and with the equivalent amount of **[Cu_2_I_2_(Apyz)]_n_** for 24 h. A colorimetric/fluorescence analysis allows to quantify the cellular viability, by means of the change of the REDOX state of the medium, in function of the cellular metabolism (or its absence), by adding resazurin. A statistical analysis was performed (1-factor ANOVA, Tukey for multiple comparisons) to find means that are significantly different from each other.

**Figure 8:**
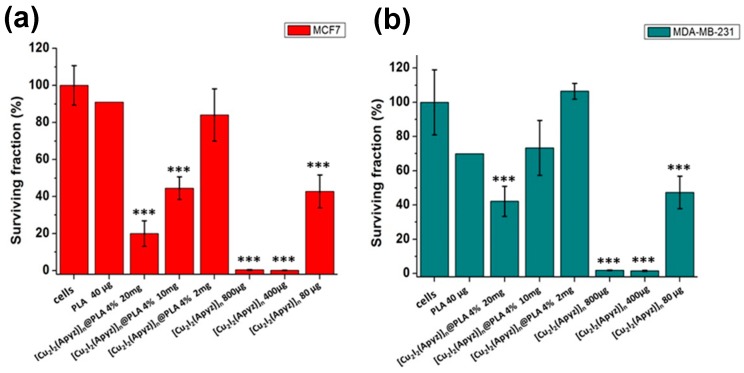
Toxicity tests of **[Cu_2_I_2_(Apyz)]_n_@PLA 4%** versus MCF7 (**a**) and MDA-MB-231 tumor cells (**b**). Variation of the surviving fraction versus amount of composite material. Statistical analysis was performed using One-way ANOVA comparing each group vs. only cell control (*** *p* < 0.001).

Taking into account that 1 mg of composite material (**[Cu_2_I_2_(Apyz)]_n_@PLA 4%**) contains 40 μg of **[Cu_2_I_2_(Apyz)]_n_** ((10.68 μg (26.70%) of Cu (I)) (Table S4). The obtained results (Figure 8) show that when 40 µg of polylactic acid are incubated with MCF7 for 24 h, the percentage of cellular survival reaches 92% compared to 70% achieved in the MDA-MB-231, not considered toxic under these conditions (Figure 8). In the same way 2 mg of **[Cu_2_I_2_(Apyz)]_n_@PLA 4%**, which contain 80 µg of [**Cu_2_I_2_(Apyz)]_n_**, show a percentage of cellular viability of 90% in MDF7 and around 100% in MDA-MB. 

Logically, 80 µg of the **[Cu_2_I_2_(Apyz)]_n_** shows a greater reduction in cellular viability of both cellular lines (Figure 8). But the most interesting thing is that when these 80 µg of **[Cu_2_I_2_(Apyz)]_n_** are introduced into the organic matrix (2mg of **[Cu_2_I_2_(Apyz)]_n_@PLA 4%**), and the composite material is created, the toxicity decreases, being even less toxic than PLA alone. So we could say that low amounts of [**Cu_2_I_2_(Apyz)]_n_**, embedded in the PLA matrix, improve its biocompatibility against the tumor cells studied.

## 4. Conclusions

The facility to nanoprocess the 2D CP **[Cu_2_I_2_(Apyz)]_n_** allows it to be integrated as nanosheets in PLA as organic matrix. The method to obtain the films is carried out by dispersion of both components at 25 °C, under ultrasounds in a chloroform suspension. In these conditions, the generated films show a high dispersion degree of the CP in the PLA. In addition, the thicknesses can be reduced up to 25 nm by using spin coating at high revolutions as surface deposition method, showing one of the first examples of ultrathin films based on coordination polymers. The films show a thermo-luminescent behavior similar to that of the isolated **[Cu_2_I_2_(Apyz)]_n_**, increasing the emission 30 times when the temperature drops from 300 to 100 K. On the other hand, the mechanical properties of the pristine PLA are altered when doping it with our CP, producing a more brittle material than the naked PLA matrix. However, despite the loss of ductility of the films their flexibility is still remarkable, so they show themselves as great candidates to be presented as biodegradable temperature sensors.

These materials lie among the first stimuli-responsive hybrid materials obtained as thin films of nanometric dimensions. The simple and cheap approach used for their preparation makes them suitable to be the base of opto-electronic devices and sensors. Finally, the biocompatibility of the PLA matrix, join with the coordination polymer could allow possible applications of these materials in vivo and simplifies their disposal and treatment, contributing to a reduction in the generation of pollutants.

## Supplementary Materials

Supporting information is available online at https://www.mdpi.com/2073-4360/11/6/1047/s1, containing the following figures and tables: Figure S1: Experimental powder X-ray diffractograms of PLA (black), **[Cu_2_I_2_(Apyz)]_n_** (red) and a **[Cu_2_I_2_(Apyz)]_n_@PLA** thin film with 30% w/w of **[Cu_2_I_2_(Apyz)]_n_** (blue), Figure S2: IR spectra of PLA (black), **[Cu_2_I_2_(Apyz)]_n_@PLA** thin films with 4% (red) and 30% w/w of **[Cu_2_I_2_(Apyz)]_n_** (blue), and **[Cu_2_I_2_(Apyz)]_n_** (green). The IR spectrum of the thin film with 1% w/w of **[Cu_2_I_2_(Apyz)]_n_** is the same observed for PLA (such low concentrations cannot be detected by IR spectroscopy), Figure S3: Thermogravimetric analyses of PLA (a) and the **[Cu_2_I_2_(Apyz)]_n_@PLA** thin film with 1% w/w of **[Cu_2_I_2_(Apyz)]_n_** (b), Figure S4: Thermogravimetric analyses of the **[Cu_2_I_2_(Apyz)]_n_@PLA** thin films with 4% (c) and 30% w/w of **[Cu_2_I_2_(Apyz)]_n_** (b), Figure S5: UV-visible spectra of naked PLA (black) and **[Cu_2_I_2_(Apyz)]_n_@PLA** composite films with 1% (red), 4% (blue) and 30% w/w of **[Cu_2_I_2_(Apyz)]_n_** (green). The thickness of the films was 40 µm. The absorbance values for λ = 750 nm were considered to calculate the transparency of the films, so that the absorption band of **[Cu_2_I_2_(Apyz)]_n_** would not interfere, Figure S6: FE-SEM images of the **[Cu_2_I_2_(Apyz)]_n_@PLA** thin films with 1% w/w (a, d, g), 4% w/w (b, e, h) and 30% w/w of **[Cu_2_I_2_(Apyz)]_n_** (c, f, i) prepared by drop casting of the corresponding suspension, Figure S7: (a-c) Backscattered electrons SEM images of the **[Cu_2_I_2_(Apyz)]_n_@PLA** thin films with 1% w/w (a), 4% w/w (b) and 30% w/w of **[Cu_2_I_2_(Apyz)]_n_** (c) prepared by drop casting of the corresponding suspension. (d-f) EDX analyses of the same **[Cu_2_I_2_(Apyz)]_n_@PLA** thin films with 1% w/w (d), 4% w/w (e) and 30% w/w of **[Cu_2_I_2_(Apyz)]_n_** (f), Figure S8: Photograph of the samples of **[Cu_2_I_2_(Apyz)]_n_@PLA** thin films used for the mechanical measurements: PLA (a), **[Cu_2_I_2_(Apyz)]_n_@PLA** thin films with 1% (b), 4% (c) and 30% w/w (d) of **[Cu_2_I_2_(Apyz)]_n_**, Figure S9: Optical microscope image of a **[Cu_2_I_2_(Apyz)]_n_@PLA**-30% sample edge with thickness measurement, Figure S10: Ultimate tensile strength for naked PLA film and **[Cu_2_I_2_(Apyz)]_n_@PLA** thin films composites with 0%, 1%, 4% and 30% (w/w) of **[Cu_2_I_2_(Apyz)]_n_**. Different amounts of PC produce slight changes in the ultimate tensile strength, Figure S11: Elastic tensile modulus (Young Modulus) for naked PLA film and thin films composites with 1%, 4% and 30% (w/w) of **[Cu_2_I_2_(Apyz)]_n_**. A small amount of CP (1%) produces a slight increase in the Elastic modulus, while an increase in the quantity of CP causes the elastic modulus to decrease, Figure S12: Elongation (%) at failure point for naked PLA film and film composites with 1%, 4% and 30% (w/w) of **[Cu_2_I_2_(Apyz)]_n._** Any amount of CP in the composite films produces a decrease of roughly 50% in the total elongation of the material. As the amount of CP increases, so does the elastic elongation, Table S1: Decomposition temperatures, maximum loss weights and residual weights of the composites **[Cu_2_I_2_(Apyz)]_n_@PLA**, with different amounts of **[Cu_2_I_2_(Apyz)]_n_**, Table S2: Tensile strength (TS), Young Modulus or Elastic Modulus (E), total elongation, plastic deformation and elastic elongation of the samples **[Cu_2_I_2_(Apyz)]_n_@PLA**, with different amounts of **[Cu_2_I_2_(Apyz)]_n_**.

## References

[1] Kwon E., Kim J., Lee K.Y., Kim T.H. (2017). Non-phase-transition luminescence mechanochromism of a copper(I) coordination polymer. Inorg. Chem..

[2] Yan Y., Chen J., Zhang N.-N., Wang M.-S., Sun C., Xing X.-S., Li R., Xu J.-G., Zheng F.-K., Guo G.-C. (2016). Grinding size-dependent mechanoresponsive luminescent Cd(II) coordination polymer. Dalton Trans..

[3] Troyano J., Perles J., Amo-Ochoa P., Martínez J.I., Gimeno M.C., Fernández-Moreira V., Zamora F., Delgado S. (2016). Luminescent thermochromism of 2D coordination polymers based on copper(I) halides with 4-hydroxythiophenol. Chem. Eur. J..

[4] Vegas V.G., Lorca R., Latorre A., Hassanein K., Gómez-García C.J., Castillo O., Somoza A., Zamora F., Amo-Ochoa P. (2017). Copper(II)-thymine coordination polymer nanoribbons as potential oligonucleotide nanocarriers. Angew. Chem. Int. Ed..

[5] Rodenas T., Luz I., Prieto G., Seoane B., Miro H., Corma A., Kapteijn F., Llabrés i Xamena F.X., Gascon J. (2015). Metal-organic framework nanosheets in polymer composite materials for gas separation. Nat. Mater..

[6] Cariati E., Lucenti E., Botta C., Giovanella U., Marinotto D., Righetto S. (2016). Cu(I) hybrid inorganic-organic materials with intriguing stimuli responsive and optoelectronic properties. Coord. Chem. Rev..

[7] Liu Y., Pan J.H., Wang N.Y., Steinbach F., Liu X.L., Caro J. (2015). Remarkably enhanced gas separation by partial self-conversion of a laminated membrane to metal-organic frameworks. Angew. Chem. Int. Ed..

[8] Xue J.-Y., Li J.-C., Li H.-X., Li H.-Y., Lang J.-P. (2016). Chan-Lam cross-coupling reactions promoted by anionic copper(I)/iodide species with cationic methyl-((pyridinyl)-pyrazolyl)pyridin-1-ium. Tetrahedron.

[9] Li J.-C., Li H.-X., Li H.-Y., Gong W.-J., Lang J.-P. (2016). Ligand coordination site-directed assembly of copper(I) iodide complexes of ((pyridyl)-1-pyrazolyl)pyridine. Cryst. Growth Des..

[10] Conesa-Egea J., Zamora F., Amo-Ochoa P. (2019). Perspectives of the smart Cu-Iodine coordination polymers: A portage to the world of new nanomaterials and composites. Coord. Chem. Rev..

[11] Dou A.N., Du Y.C., Chen Q.L., Luo K.L., Zhang C., Zhu A.X., Li Q.X. (2016). 3D Luminescent copper(I) iodide coordination polymer based on Cu_4_I_4_ clusters and an ethyl-bridging bis(triazole) ligand. Zeitschrift für Anorganische und Allgemeine Chemie.

[12] Demir S., Cepni H.M., Bilgin N., Holynska M., Yilmaz F. (2016). Metal-organic frameworks based on copper(I) iodide and pyridine-3,5-dicarboxylic acid: Synthesis, crystal structures and luminescent properties. Polyhedron.

[13] Zhao J., Wang Y.-N., Dong W.-W., Wu Y.-P., Li D.-S., Zhang Q.-C. (2016). A robust luminescent Tb(III)-MOF with Lewis basic pyridyl sites for the highly sensitive detection of metal ions and small molecules. Inorg. Chem..

[14] Song Y., Fan R.Q., Wang P., Wang X.M., Gao S., Du X., Yang Y.L., Luan T.Z. (2015). Copper(I)-iodide based coordination polymers: Bifunctional properties related to thermochromism and PMMA-doped polymer film materials. J. Mater. Chem. C.

[15] Benito Q., Le Goff X.-F., Nocton G., Fargues A., Garcia A., Berhault A., Kahlal S., Saillard J.Y., Martineau C., Trebosc J. (2015). Geometry flexibility of copper iodide clusters: Variability in luminescence thermochromism. Inorg. Chem..

[16] Zhao C.-W., Ma J.-P., Liu Q.-K., Wang X.-R., Liu Y., Yang J., Yang J.-S., Dong Y.-B. (2016). An in situ self-assembled Cu_4_I_4_-MOF-based mixed matrix membrane: A highly sensitive and selective naked-eye sensor for gaseous HCl. Chem. Commun..

[17] Hassanein K., Conesa-Egea J., Delgado S., Castillo O., Benmansour S., Martinez J.I., Abellan G., Gomez-Garcia C.J., Zamora F., Amo-Ochoa P. (2015). Electrical conductivity and strong luminescence in copper iodide double chains with isonicotinato derivatives. Chem. Eur. J..

[18] Yan Y., Zhang N.-N., Li R., Xu J.-G., Lu J., Zheng F.-K., Guo G.-C. (2017). Coordination polymers with grinding-size-dependent mechanoresponsive luminescence induced by π···π stacking interactions. Eur. J. Inorg. Chem..

[19] Conesa-Egea J., Gallardo-Martínez J., Delgado S., Martínez J.I., Gonzalez-Platas J., Fernández-Moreira V., Rodríguez-Mendoza U.R., Ocón P., Zamora F., Amo-Ochoa P. (2017). Multistimuli response micro- and nanolayers of a coordination polymer based on cu_2_i_2_ chains linked by 2-aminopyrazine. Small.

[20] Amo-Ochoa P., Hassanein K., Gomez-Garcia C.J., Benmansour S., Perles J., Castillo O., Martinez J.I., Ocon P., Zamora F. (2015). Reversible stimulus-responsive Cu(I) iodide pyridine coordination polymer. Chem. Commun..

[21] Rodriguez-San-Miguel D., Amo-Ochoa P., Zamora F. (2016). MasterChem: Cooking 2D-polymers. Chem. Commun..

[22] Conesa-Egea J., Nogal N., Martínez J.I., Fernández-Moreira V., Rodríguez-Mendoza U.R., González-Platas J., Gómez-García C.J., Delgado S., Zamora F., Amo-Ochoa P. (2018). Smart composite films of nanometric thickness based on copper-iodine coordination polymers. Toward sensors. Chem. Sci..

[23] Chuayjuljit S., Wongwaiwattanakul C., Chaiwutthinan P., Prasassarakich P. (2017). Biodegradable poly(lactic acid)/poly(butylene succinate)/wood flour composites: Physical and morphological properties. Polym. Compos..

[24] Liu W., Fang Y., Wei G.Z., Teat S.J., Xiong K., Hu Z., Lustig W.P., Li J. (2015). A family of highly efficient CuI-based lighting phosphors prepared by a systematic, bottom-up synthetic approach. J. Am. Chem. Soc..

[25] Horcas I., Fernández R., Gómez-Rodríguez J.M., Colchero J., Gómez-Herrero J., Baro A.M. (2007). WSXM: A software for scanning probe microscopy and a tool for nanotechnology. Rev. Sci. Instr..

[26] Troyano J., Castillo O., Martínez J.I., Fernández-Moreira V., Ballesteros Y., Maspoch D., Zamora F., Delgado S. (2018). Reversible thermochromic polymeric thin films made of ultrathin 2D crystals of coordination polymers based on copper(I)-thiophenolates. Adv. Funct. Mater..

[27] Denny M.S., Cohen S.M. (2015). In situ modification of metal-organic frameworks in mixed-matrix membranes. Angew. Chem. Int. Ed..

[28] Monzón-Hierro T., Sanchiz J., González-Pérez S., González-Díaz B., Holinski S., Borchert D., Hernández-Rodríguez C., Guerrero-Lemus R. (2015). A new cost-effective polymeric film containing an Eu(III) complex acting as UV protector and down-converter for Si-based solar cells and modules. Sol. Energy Mater. Sol. Cells.

[29] González-Pérez S., Sanchiz J., González-Díaz B., Holinski S., Borchert D., Hernández-Rodríguez C., Guerrero-Lemus R. (2015). Luminescent polymeric film containing an Eu(III) complex acting as UV protector and down-converter for Si-based solar cells and modules. Surf. Coat. Technol..

[30] Kräuter G., Schumacher A., Gösele U. (1998). Low temperature silicon direct bonding for application in micromechanics: Bonding energies for different combinations of oxides. Sens. Act. A Phys..

