# Supplementary material for: Micro and Nano Smart Composite Films Based on Copper-Iodine Coordination Polymer as Thermochromic Biocompatible Sensors
Source: Polymers (Basel). 2019 Jun 15;11(6):1047. doi: 10.3390/polym11061047 (PMC6630835; doi:10.3390/polym11061047)
Supplement: Supplementary file 1 [file polymers-11-01047-s001.pdf]

Micro and Nano Smart Composite Films based on Copper-Iodine Coordination Polymer as Thermochromic Biocompatible Sensors.

Javier Conesa-Egea, Alberto Moreno-Vázquez, Vanesa Fernández-Moreira, Yolanda Ballesteros, Milagros Castellanos, Félix Zamora, and Pilar Amo-Ochoa*

Supporting Information

S1. Structural and thermal characterization of **$[Cu_2I_2(Apyz)]_n$@PLA** nanosheets. S2

S2. Transparency of **$[Cu_2I_2(Apyz)]_n$@PLA** nanosheets S5

S3. Morphological characterization of **$[Cu_2I_2(Apyz)]_n$@PLA** thin films. S6

S4. Mechanical testing of **$[Cu_2I_2(Apyz)]_n$@PLA** films. S7

S1. Structural and thermal characterization of $[Cu_2I_2(Apyz)]_n$@PLA nanosheets.

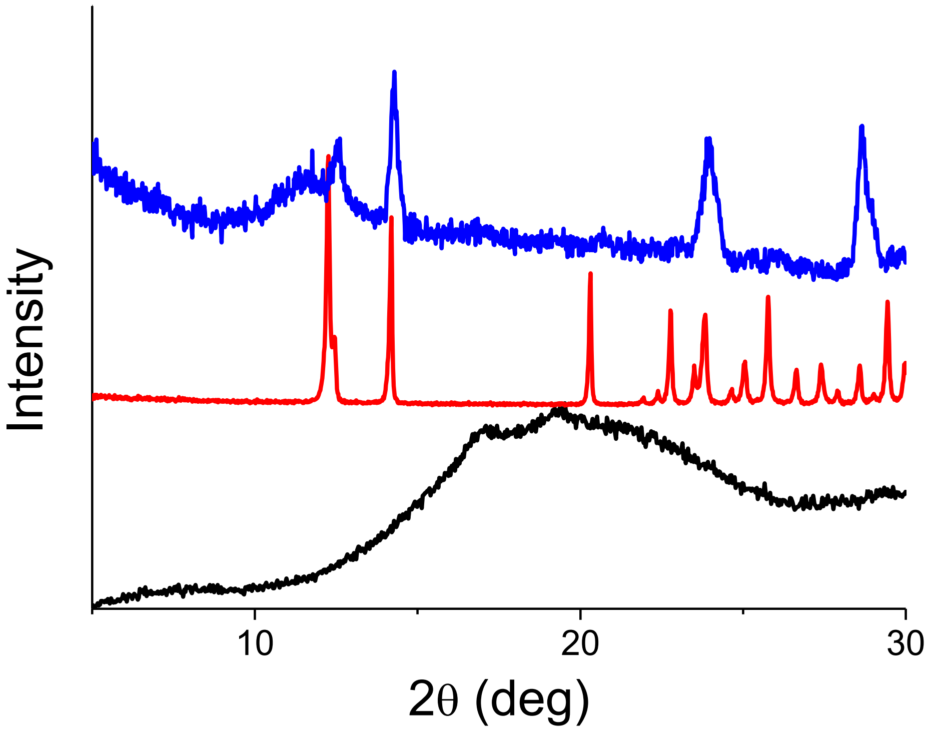

Figure S1. Experimental powder X-ray diffractograms of PLA (black), **$[Cu_2I_2(Apyz)]_n$** (red) and a **$[Cu_2I_2(Apyz)]_n$@PLA** thin film with 30% w/w of **$[Cu_2I_2(Apyz)]_n$** (blue).

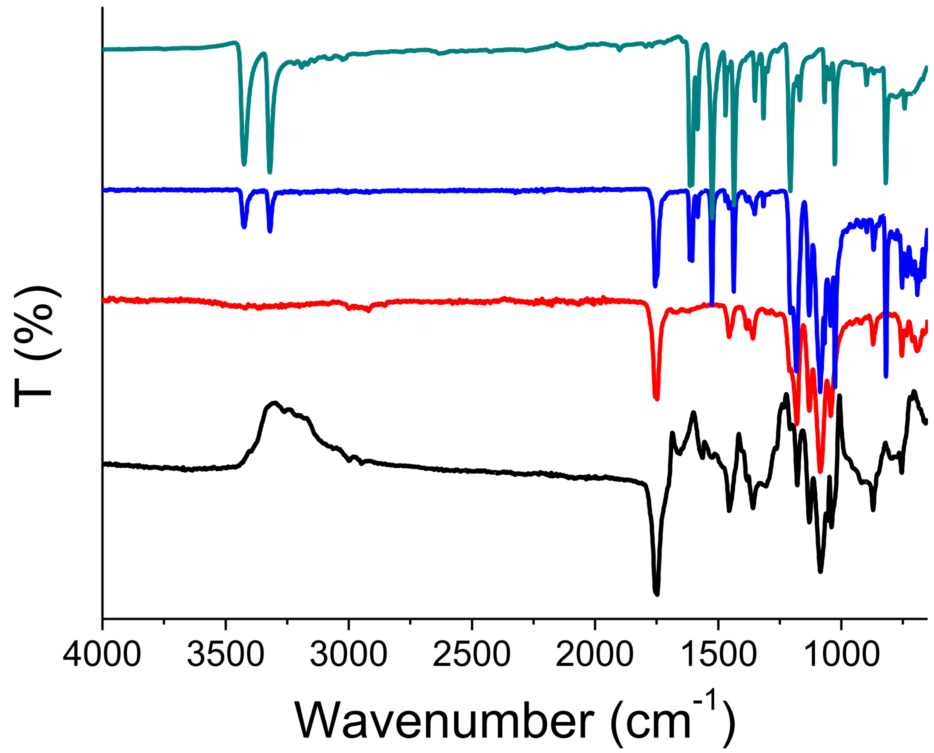

Figure S2. IR spectra of PLA (black), **$[Cu_2I_2(Apyz)]_n$@PLA** thin films with 4% (red) and 30% w/w of **$[Cu_2I_2(Apyz)]_n$** (blue), and **$[Cu_2I_2(Apyz)]_n$** (green). The IR spectrum of the thin film with 1% w/w of **$[Cu_2I_2(Apyz)]_n$** is the same observed for PLA (such low concentrations cannot be detected by IR spectroscopy).

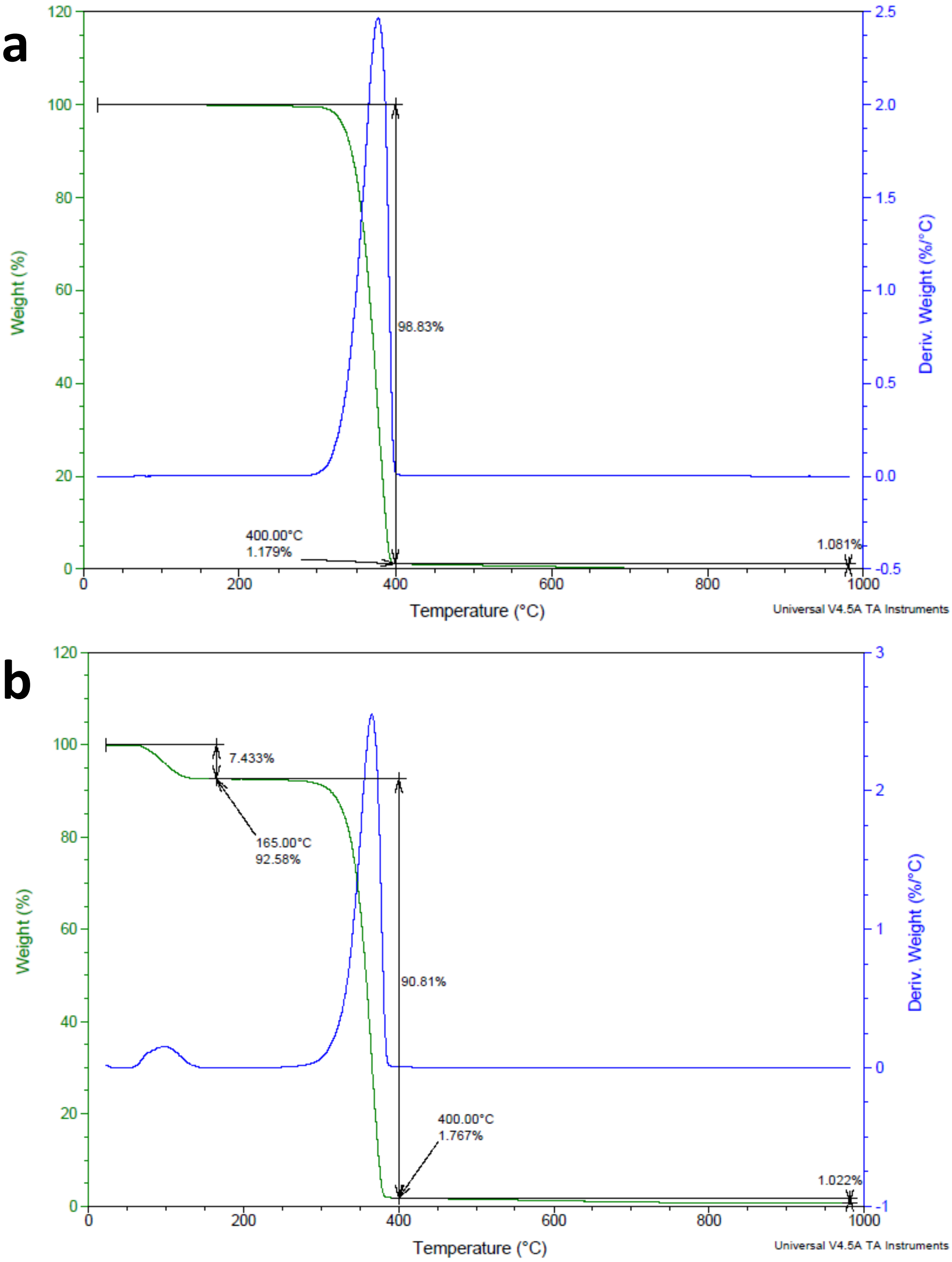

Figure S3. Thermogravimetric analyses of PLA (a) and the **$[Cu_2I_2(Apyz)]_n$@PLA** thin film with 1% w/w of **$[Cu_2I_2(Apyz)]_n$** (b).

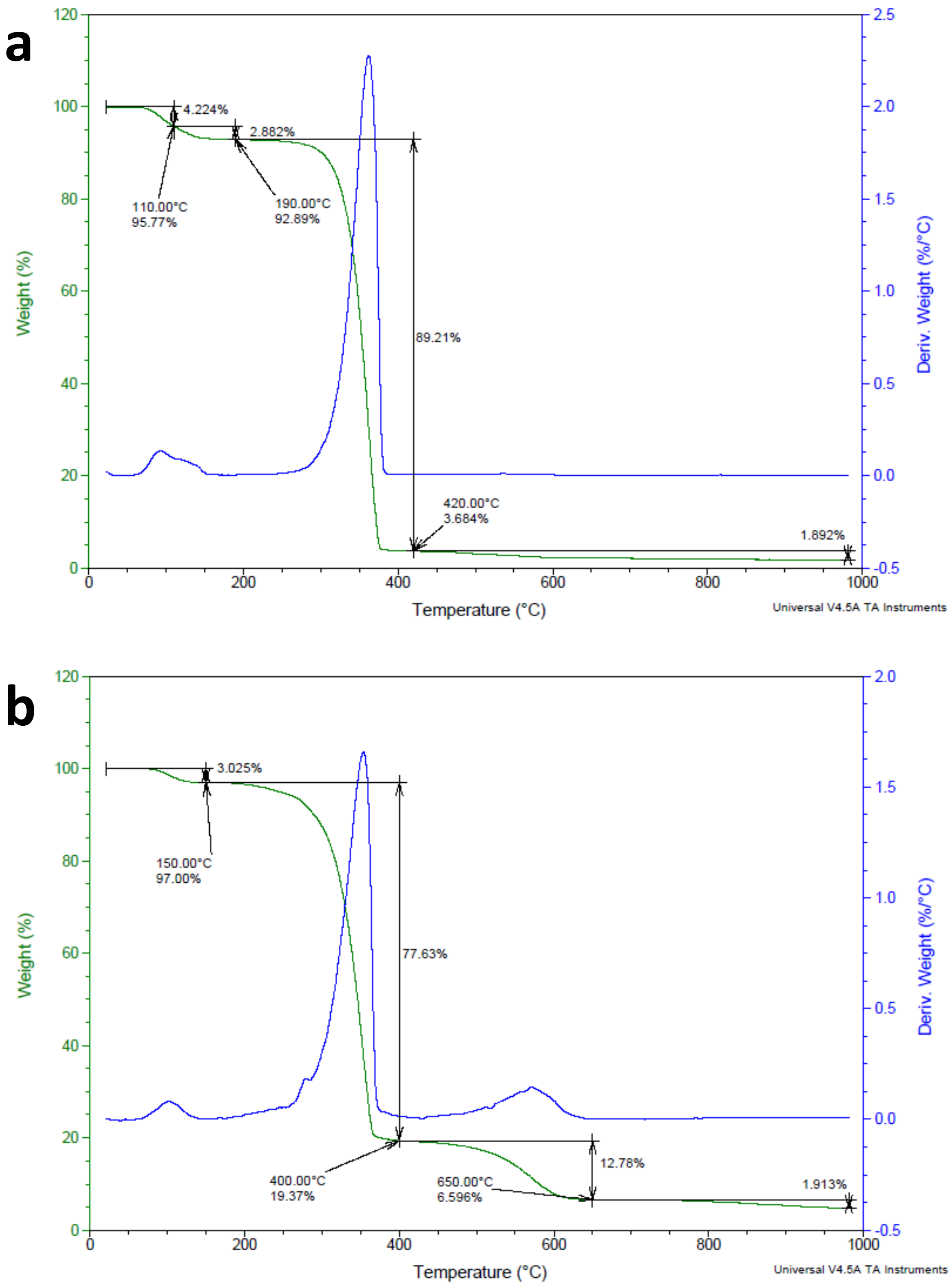

Figure S4. Thermogravimetric analyses of the **[Cu$_2$I$_2$(Apyz)]$_n$@PLA** thin films with 4% (c) and 30% w/w of **[Cu$_2$I$_2$(Apyz)]$_n$** (b).

Table S1. Decomposition temperatures, maximum loss weights and residual weights of the composites **[Cu_2I_2(Apyz)]$_n$@PLA**, with different amounts of **[Cu_2I_2(Apyz)]$_n$**.

Sample	$T_{5\%}$ (°C)	T_{max} (°C)	D_{max} (%/°C)	W_R (%)
PLA	334	378	2.466	0.098
[Cu_2I_2(Apyz)]$_n$@PLA 1%	105	365	2.553	0.746
[Cu_2I_2(Apyz)]$_n$@PLA 4%	118	362	2.276	1.792
[Cu_2I_2(Apyz)]$_n$@PLA 30%	245	354	1.658	4.683

S2. Transparency of [Cu_2I_2(Apyz)]$_n$@PLA nanosheets

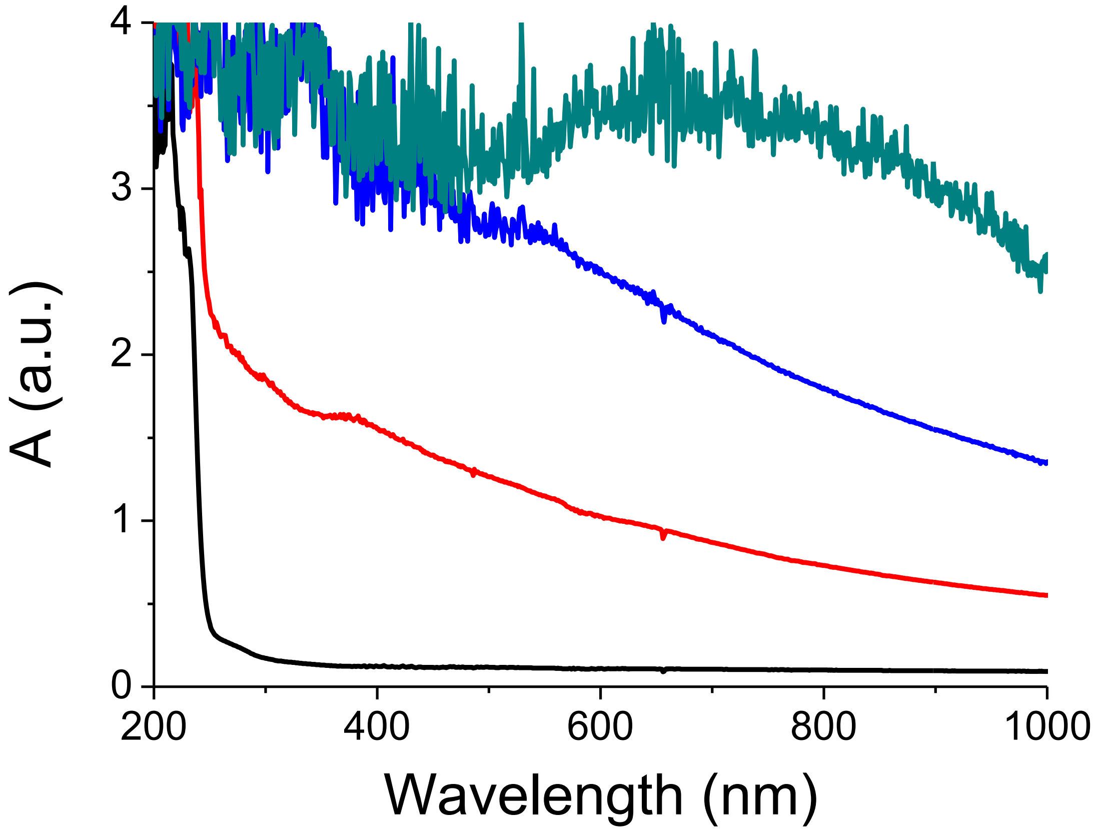

Figure S5. UV-visible spectra of naked PLA (black) and **[Cu_2I_2(Apyz)]$_n$@PLA** composite films with 1% (red), 4% (blue) and 30% w/w of **[Cu_2I_2(Apyz)]$_n$** (green). The thickness of the films was 40 µm. The absorbance values for λ = 750 nm were considered to calculate the transparency of the films, so that the absorption band of **[Cu_2I_2(Apyz)]$_n$** would not interfere.

S3. Morphological characterization of $[Cu_2I_2(Apyz)]_n$@PLA thin films.

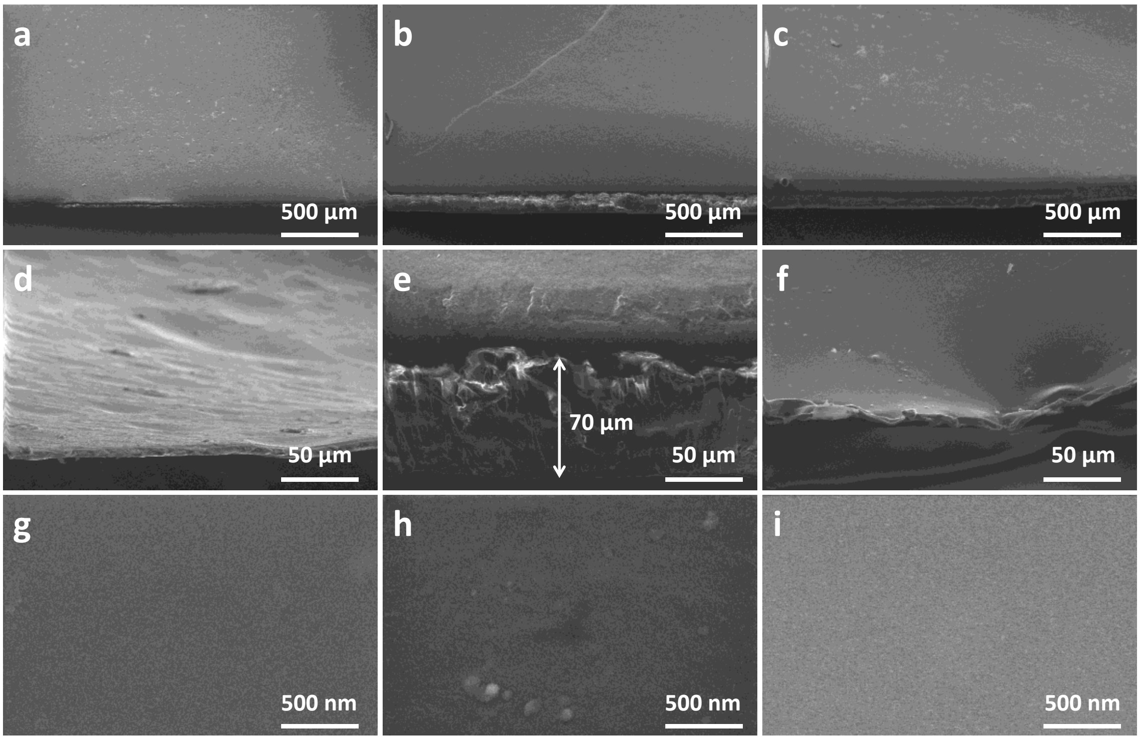

Figure S6. FE-SEM images of the **$[Cu_2I_2(Apyz)]_n$@PLA** thin films with 1% w/w (a, d, g), 4% w/w (b, e, h) and 30% w/w of **$[Cu_2I_2(Apyz)]_n$** (c, f, i) prepared by drop casting of the corresponding suspension.

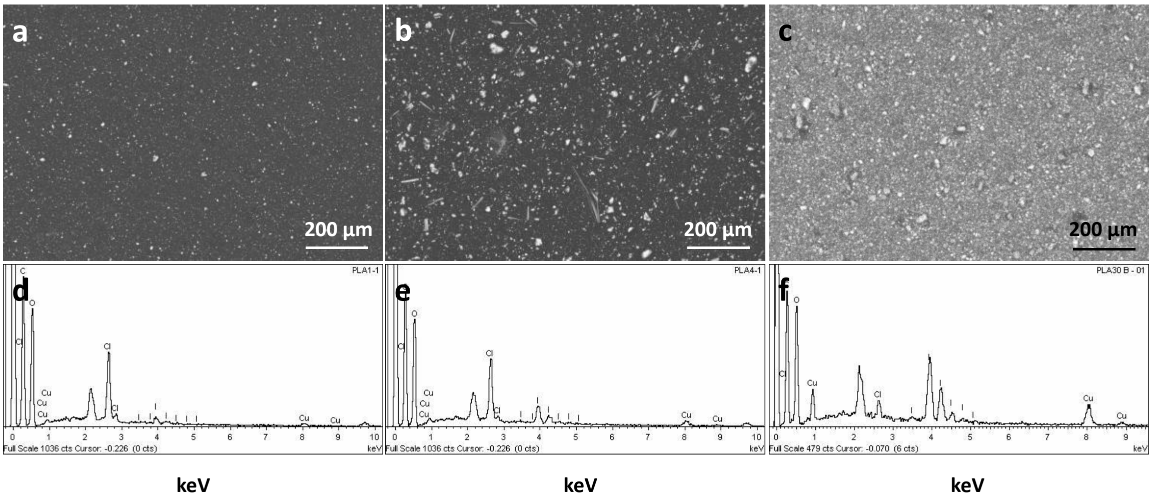

Figure S7. (a-c) Backscattered electrons SEM images of the **$[Cu_2I_2(Apyz)]_n$@PLA** thin films with 1% w/w (a), 4% w/w (b) and 30% w/w of **$[Cu_2I_2(Apyz)]_n$** (c) prepared by drop casting of the corresponding suspension. (d-f) EDX analyses of the same **$[Cu_2I_2(Apyz)]_n$@PLA** thin films with 1% w/w (d), 4% w/w (e) and 30% w/w of **$[Cu_2I_2(Apyz)]_n$** (f).

S4. Mechanical testing of $[Cu_2I_2(Apyz)]_n$@PLA thin films.

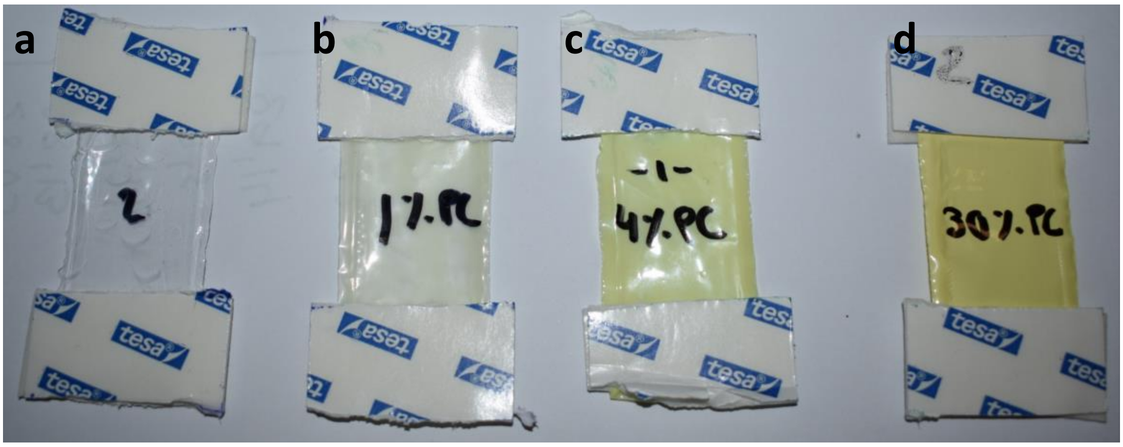

Figure S8. Photograph of the samples of **$[Cu_2I_2(Apyz)]_n$@PLA** thin films used for the mechanical measurements: PLA (a), **$[Cu_2I_2(Apyz)]_n$@PLA** thin films with 1% (b), 4% (c) and 30% w/w (d) of **$[Cu_2I_2(Apyz)]_n$**.

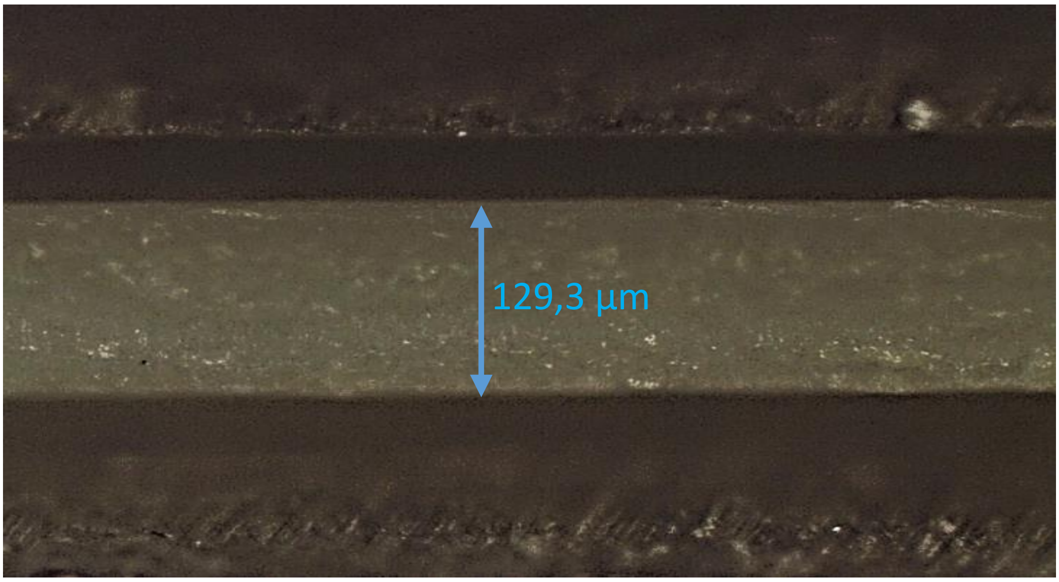

Figure S9. Optical microscope image of a **$[Cu_2I_2(Apyz)]_n$@PLA**-30% sample edge with thickness measurement.

Table S2. Tensile strength (TS), Young Modulus or Elastic Modulus (E), total elongation, plastic deformation and elastic elongation of the samples **$[Cu_2I_2(Apyz)]_n$@PLA**, with different amounts of **$[Cu_2I_2(Apyz)]_n$**.

Sample	TS (MPa)	E (MPa)	Total Elongation (%)	Plastic Deformation (%)	Elastic Elongation (%)
PLA	40.9 ± 1.1	948 ± 42	33.0 ± 4.2	18.9 ± 1.6	14.1 ± 2.8
$[Cu_2I_2(Apyz)]_n$@PLA 1%	44.1 ± 3,1	1081 ± 24	16.3 ± 1.0	9.2 ± 4.0	7.2 ± 3.0
$[Cu_2I_2(Apyz)]_n$@PLA 4%	40.9 ± 1.0	902 ± 29	15.3 ± 1.8	6.1 ± 0.4	9.2 ± 1.3
$[Cu_2I_2(Apyz)]_n$@PLA 30%	38.5 ± 1.1	786 ± 72	17.2 ± 4.3	3.6 ± 0.4	13.7 ± 3.6

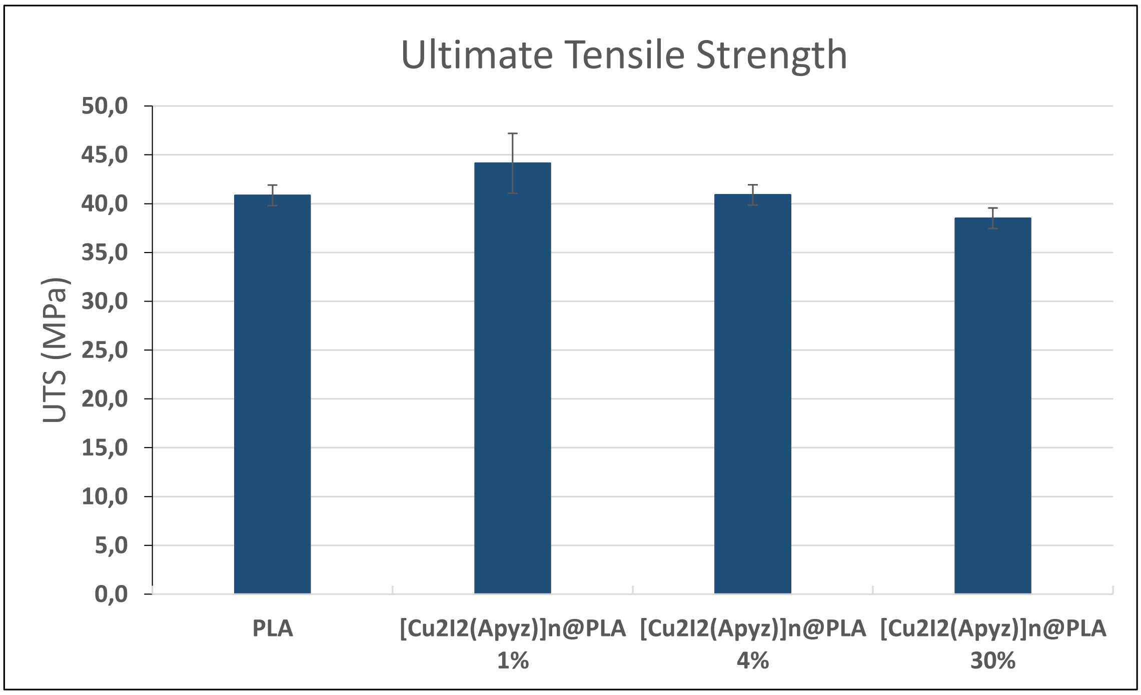

Figure S10. Ultimate tensile strength for naked PLA film and **$[Cu_2I_2(Apyz)]_n$@PLA** thin films composites with 0%, 1%, 4% and 30% (w/w) of **$[Cu_2I_2(Apyz)]_n$**. Different amounts of PC produce slight changes in the ultimate tensile strength.

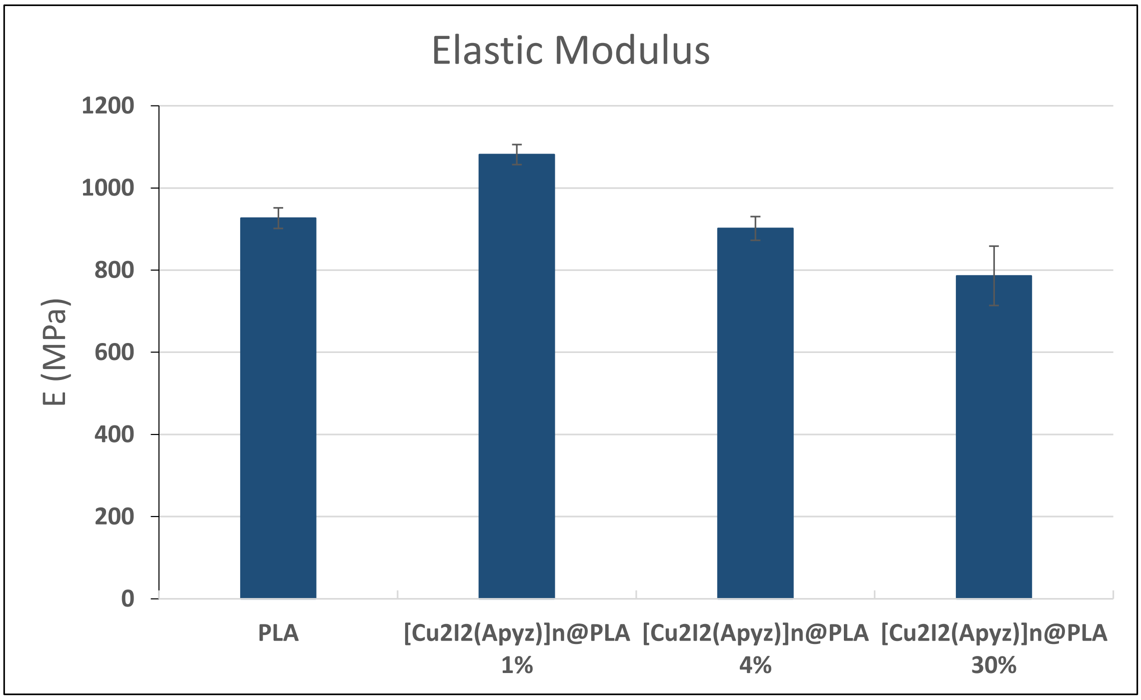

Figure S11. Elastic tensile modulus (Young Modulus) for naked PLA film and thin films composites with 1%, 4% and 30% (w/w) of **$[Cu_2I_2(Apyz)]_n$**. A small amount of CP (1%) produces a slight increase in the Elastic modulus, while an increase in the quantity of CP causes the elastic modulus to decrease.

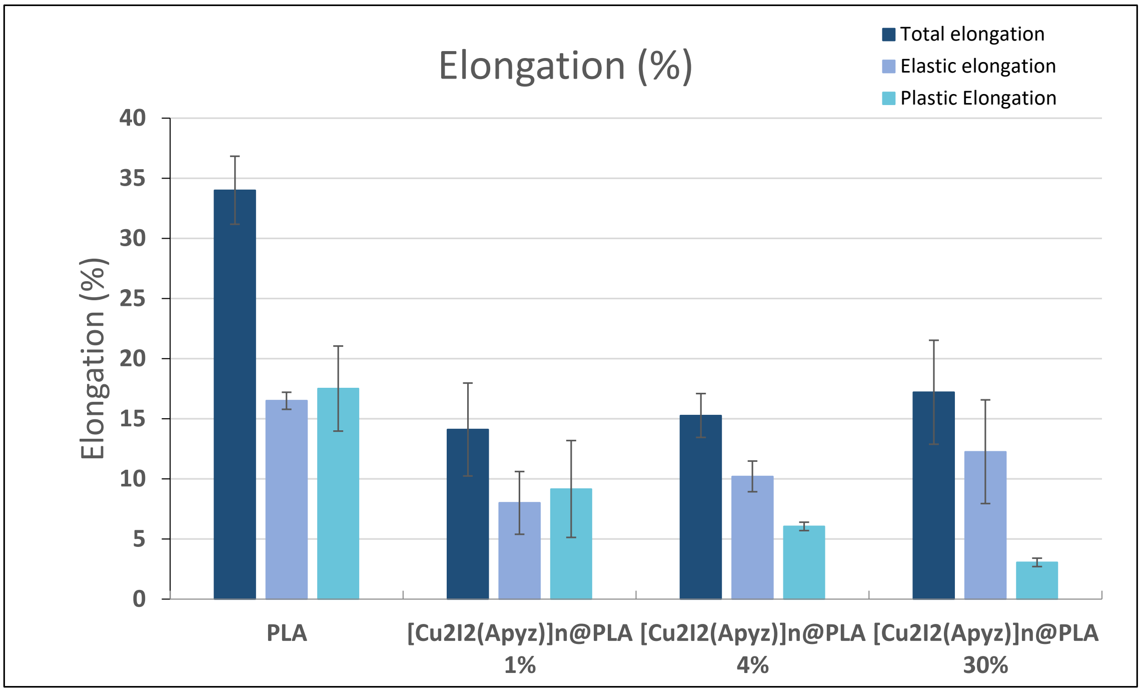

Figure S12. Elongation (%) at failure point for naked PLA film and film composites with 1%, 4% and 30% (w/w) of **$[Cu_2I_2(Apyz)]_n$**. Any amount of CP in the composite films produces a decrease of roughly 50% in the total elongation of the material. As the amount of CP increases, so does the elastic elongation.

Measurement parameters: To measure the roughness, a standard sensor has been used, which moves at a speed of 0.3 mm / s on the surface. For each sample, 3 measurements were taken in different areas of the sheet, always on the same side (upper face) with an evaluation length of 4.0 mm, with a sampling length of 0.8 mm (the one indicated by the UNE-EN ISO 4288 standard): 1998 for roughness values Ra between 0.2 and 2). For each sample, the values of Ra (arithmetic mean roughness), Rq (mean square roughness) and roughness profile were obtained (table S3 and figure S14).

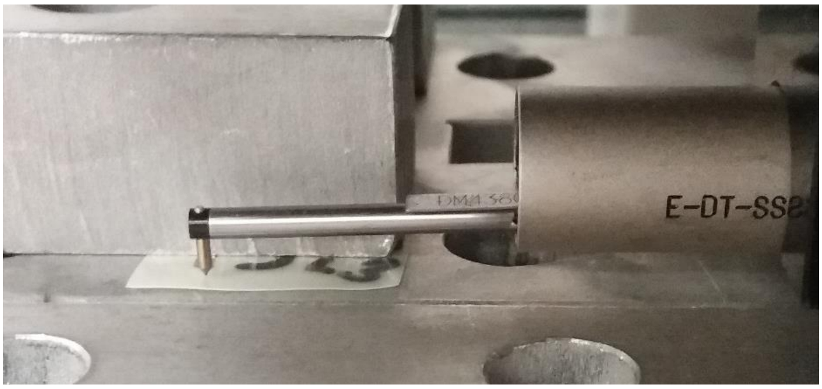

Figure S13: Image of the device for measuring the roughness of the films.

Table S3: Roughness results obtained for the three composite materials.

**Roughness**	**$[Cu_2I_2(Apyz)]_n$@PLA 1%**	**$[Cu_2I_2(Apyz)]_n$@PLA 4%**	**$[Cu_2I_2(Apyz)]_n$@PLA 30%**
**Ra (µm)**	**0.24**	**0.23**	**0.20**
**Rq (µm)**	**0.31**	**0.34**	**0.29**

Figure S14: Roughness profiles of $[Cu_2I_2(Apyz)]_n$@PLA.

$[Cu_2I_2(Apyz)]_n$@PLA 1%

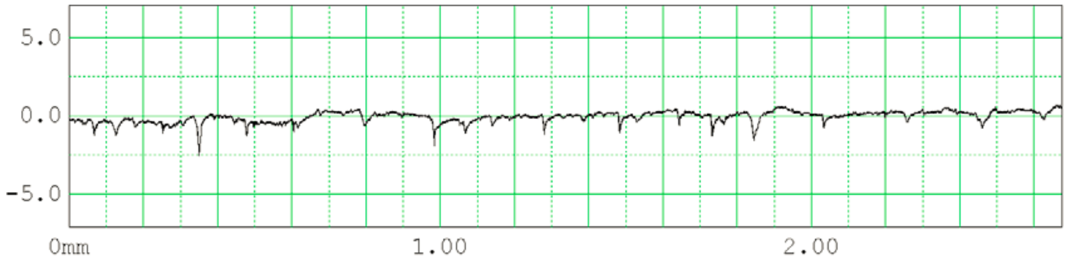

$[Cu_2I_2(Apyz)]_n$@PLA 4%

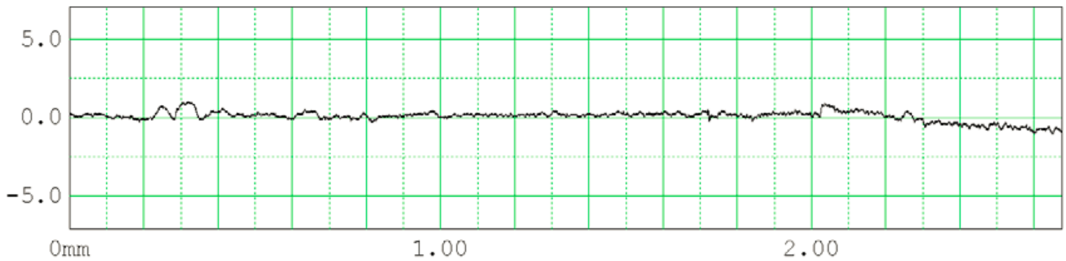

$[Cu_2I_2(Apyz)]_n$@PLA 30%

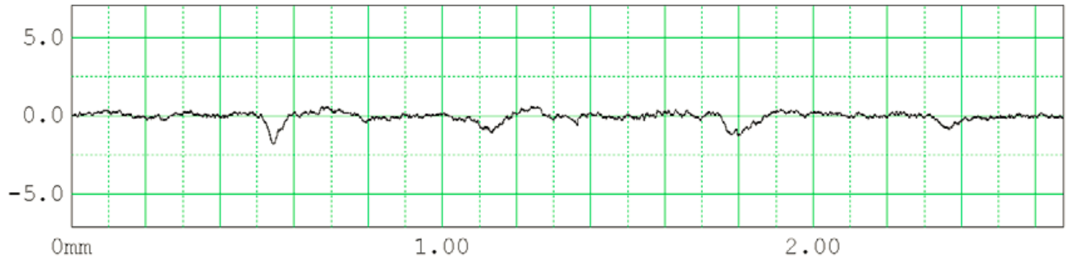

Table S4: Equivalence between samples of $[Cu_2I_2 (Apyz)]_n$ and $[Cu_2I_2 (Apyz)]_n$ @ PLA-4% in amount of copper:

**$[Cu_2I_2(Apyz)]_n$@PLA-4%** (µg)	**PLA** mass in **$[Cu_2I_2(Apyz)]_n$@PLA-4%** (µg)	**$[Cu_2I_2(Apyz)]_n$** mass in **$[Cu_2I_2(Apyz)]_n$@PLA-4%** (µg)	Cu^+ mass in **$[Cu_2I_2(Apyz)]_n$@PLA-4%** (µg)
20000	19200	800	213.6
10000	9600	400	106.8
2000	1920	80	21.36
